# Accelerated evidence synthesis in orthopaedics—the roles of natural language processing, expert annotation and large language models

**DOI:** 10.1186/s40634-023-00662-4

**Published:** 2023-09-28

**Authors:** Bálint Zsidai, Janina Kaarre, Ann-Sophie Hilkert, Eric Narup, Eric Hamrin Senorski, Alberto Grassi, Olufemi R. Ayeni, Volker Musahl, Christophe Ley, Elmar Herbst, Michael T. Hirschmann, Sebastian Kopf, Romain Seil, Thomas Tischer, Kristian Samuelsson, Robert Feldt, ESSKA Artificial Intelligence Working Group

**Affiliations:** 1 Sahlgrenska Sports Medicine Center, Gothenburg, Sweden; 2 Department of Orthopaedics, Institute of Clinical Sciences, Sahlgrenska Academy, University of Gothenburg, Gothenburg, Sweden; 3 Department of Orthopaedic Surgery, UPMC Freddie Fu Sports Medicine Center, University of Pittsburgh, Pittsburgh, USA; 4 Department of Computer Science and Engineering, Chalmers University of Technology, Gothenburg, Sweden; 5 Medfield Diagnostics AB, Gothenburg, Sweden; 6 Department of Health and Rehabilitation, Institute of Neuroscience and Physiology, Sahlgrenska Academy, University of Gothenburg, Gothenburg, Sweden; 7 Sportrehab Sports Medicine Clinic, Gothenburg, Sweden; 8 IIa Clinica Ortopedica E Traumatologica, IRCCS Istituto Ortopedico Rizzoli, Bologna, Italy; 9 Division of Orthopaedic Surgery, Department of Surgery, McMaster University, Hamilton, Canada; 10 Department of Mathematics, University of Luxembourg, Esch-Sur-Alzette, Luxembourg; 11 Department of Trauma, Hand and Reconstructive Surgery, University Hospital Münster, Münster, Germany; 12 Department of Orthopedic Surgery and Traumatology, Head Knee Surgery and DKF Head of Research, Kantonsspital Baselland, 4101 Bruderholz, Bottmingen Switzerland; 13 Center of Orthopaedics and Traumatology, University Hospital Brandenburg a.d.H., Brandenburg Medical School Theodor Fontane, 14770 Brandenburg, Germany; 14 Faculty of Health Sciences Brandenburg, Brandenburg Medical School Theodor Fontane, 14770 Brandenburg, Germany; 15 Department of Orthopaedic Surgery, Centre Hospitalier Luxembourg and Luxembourg Institute of Health, Luxembourg, Luxembourg; 16 Clinic for Orthopaedics and Trauma Surgery, Malteser Waldkrankenhaus St. Marien, Erlangen, Germany; 17 Department of Orthopaedics, Sahlgrenska University Hospital, Mölndal, Sweden

**Keywords:** NLP, LLM, Evidence synthesis, Foundation models, AI, ML, Generative AI, Sports Medicine

In an era of electronical medical records, rapidly expanding publication rates of medical knowledge, and large-scale registries, orthopaedics is in a dire need of innovative approaches to facilitate the adoption of the latest knowledge in clinical practice. While machine learning (ML) has been heralded as one solution to many research tasks hampered by previous technological limitations [12], there is an increasing need to direct our attention towards subdomains of ML that are convenient for the extraction of meaningful clinical information stored in medical records. We believe natural language processing (NLP) to be one such domain of ML, with an immense future potential to catalyse rate-limiting steps in orthopaedic research.

## Fundamental concepts

Natural language processing is a ML-based tool that involves quantitative encoding of information derived from human language. Data generated from speech- and text-processing NLP algorithms can be used to solve a variety of tasks with broad applications in medical practice and research. Due to limited examples of NLP-based research in orthopaedics [3, 15], commonly used NLP tasks are best illustrated with examples of their potential applications across medical fields:

- *Text classification* – Categorisation and clustering of scientific articles based on level of evidence and/or sub-topics, detected using abstract screening for relevant terms.
- *Information extraction* – Identification of information related to patients, interventions, comparisons, and outcome variables (PICO elements) [2] from electronic medical records (EMR) and publications using, for example, named entity recognition (NER).
- *Question answering* – Automated responses to frequently asked questions with a custom medical knowledge base used to generate conversational layers.
- *Sentiment analysis* – Assessment of the emotions and opinions of patients about a medical service based on analysis of the affective qualities of written reviews [4].
- *Summarization* – Abstraction of a large volume of medical evidence to generate a short summary with essential and easy to understand information for patients.

Understanding of the inner workings and performance of ML models are key steps in identifying applications for NLP in orthopaedic research [10]. Accuracy (closeness), precision (exactness), recall (positive predictive value) and the *F*_1_ score (a combination of precision and recall) are key metrics used in the evaluation and interpretation of NLP models.

## Barriers to automated data extraction

While there is no shortage of available data for orthopaedic research, a major barrier to the accessibility of data is due to its storage as unstructured text. A previously published editorial outlined the discrepancy between the publication rate of primary research articles and the synthesis of up-to-date evidence in the form of systematic reviews and meta-analyses [18]. Consequently, the concept of living evidence synthesis was proposed to tackle this problem, which largely relies on NLP for near real-time extraction and compilation of relevant medical data. Additionally, the widespread adoption of EMRs by healthcare systems across the globe provides a wealth of untapped medical knowledge in the form of deidentified patient data. Unfortunately, the lack of standardization and consistency in medical documentation poses difficulties for the automated extraction of relevant and accurate information. Early results show improved performance in clinical predictions when structured EMR data is complemented with NLP analysis of unstructured EMR text [13]. While both supervised [9] and unsupervised [1] ML approaches are available for NLP, information extraction from medical text are likely to benefit from context-specific interpretation. Problematically, medical text is heterogeneous in structure and style, with a vast possibility of syntactic and semantic variability (such as abbreviations), which in turn leads to ambiguous interpretation by both humans and computers [7]. The design of automated frameworks for reliable entity and pattern-recognition in such complex environments is a critical challenge to overcome. Supervised ML methods using labelling instructions agreed upon by domain experts may reduce annotation errors, and lead to a higher quality of information extraction from context-specific text data [11]. For example, a panel of experts in ACL surgery would have the possibility to develop labelling instructions and benchmarks for extracting data from medical records regarding postoperative outcomes after ACL reconstruction. The panel would need to reach a consensus on the essential components to label, such as graft tunnel placement, graft choice and thickness, presence or absence of anterolateral augmentation, among others. Labelling instructions would thereby help establish benchmarks for consistency and reproducibility in NLP-driven research, and maximize the quality of evidence synthesis across the international orthopaedic community. It is important to point out that the clinical utility of AI systems depends heavily on the magnitude and quality of training data, which leads to concern regarding the ethical and secure access to patient information. Consequently, future efforts will also require carefully planned regulatory supervision to safeguard the national and international distribution of patient data extracted from medical records with NLP [5].

## Condition-specific annotation and NLP frameworks

**Fig. 1:**
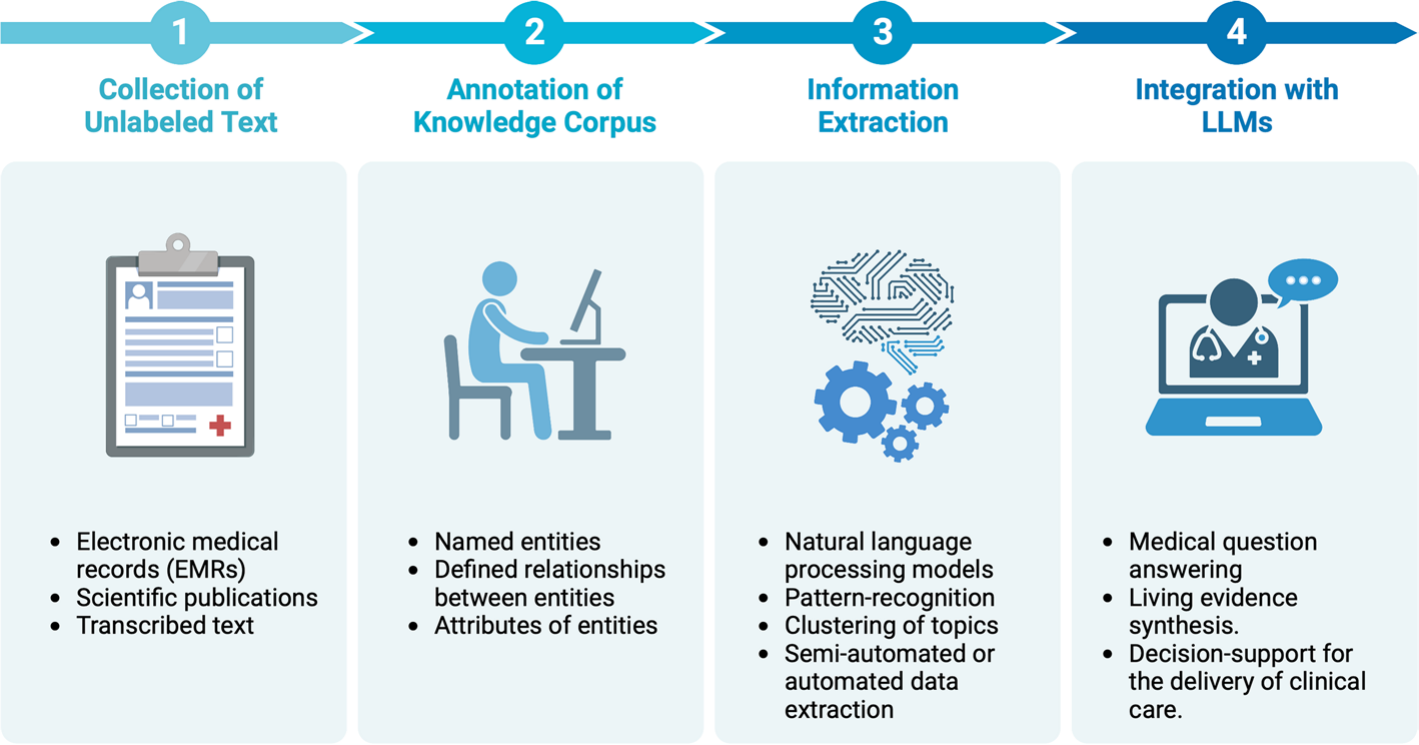
Key steps in the collaborative collection, annotation, and extraction of medical data for living evidence synthesis and integration with LLMs

The use of standardized knowledge bases is essential for the design and implementation of NLP algorithms designed for specific research purposes. We believe the next step towards solving the challenges associated with information extraction is to establish comprehensive knowledge-base of annotated disease- or injury-specific medical text. This idea rests on the principle that an NLP model is more likely to perform well when trained on a body of domain-specific information, with expert-level annotation and abstraction of the key element in the text, even if it has been pre-trained for general language understanding. A recent study of biomedical image analysis determined that improvements in labelling instructions have an immense impact on the interrater variability in the quality and consistency of annotations, and consequently, on the performance of the final algorithm [11]. Similarly, clearly formulated instructions established by domain experts may mitigate some of the errors pervasive to labelling due to time pressure, variability in motivation, differences in knowledge or style, and interpretation of the text [7]. Importantly, expert annotation of training data for a given area of orthopaedics should focus on creating a consistent and replicable framework for NLP application, which clearly distinguishes entities, relationships between different entities, and multiple attributes specific to individual entities [17]. This approach could then be considered a standard operating procedure for reliable and accurate extraction of essential medical information from medical charts and primary research articles (Fig. 1). Consequently, we propose the creation of annotated collections of scientific text based on expert consensus, specific to musculoskeletal conditions affecting the spine, shoulder, hip, knee, and ankle joints, to expedite data extraction and the synthesis of up-to-date evidence using NLP tools. Due to the inherent complexity of the task, the annotation of medical knowledge will require the interdisciplinary cooperation of healthcare professionals, linguists, and computer scientists.

## The potential of large language models

Over the recent year, large language models (LLMs), such as GPT-4 [8], Med-PaLM 2 [14], among others, showcased the revolutionary impact of medical question-answering with generative AI (GAI) on the healthcare sector. Expert-annotated, foundational datasets designed for NLP tasks may be integrated with LLMs to perform a variety of tasks, expediting both orthopaedic research, the appraisal of existing evidence and the delivery of orthopaedic care in the clinic. Annotation of important clinical concepts and their relations in EHRs, operative notes, radiology notes, and research studies based on semantic similarity may be used to train LLMs for performing clinically useful tasks with high efficiency and accuracy [16]. Additionally, GAI may be applied in a broader sense, with the capability to interpret multimodal, domain-specific information, including labelled or unlabelled medical images, patient interviews and patient reported outcome data in the context of complex clinical scenarios [6]. Harnessing the potential of LLMs and GAI may catalyse the development of clinical decision-support tools to optimize the quality of treatment for patients with orthopaedic conditions. Such endeavours require strict emphasis on the quality of data used for training foundational datasets, which necessitates expert consensus to lay out standards for the information used to design systems with advanced medical reasoning capabilities.

## Conclusion

We believe the adoption of NLP frameworks to be one of the key steps in the evolution of medical data extraction and evidence-synthesis. There is currently a need for innovative solutions to obtain meaningful information from the growing availability of structured and unstructured medical text, with the goal to improve the quality of patient care. Considering the immense potential in the clinical and research setting, there is a growing need for the dedicated training of healthcare professionals in the fundamental concepts and applications of AI. The annotation of condition-specific training data and design of efficient NLP pipelines are complex tasks, which require close collaboration between the healthcare and technology sectors to establish high-quality and scalable systems despite existing disparities across the global healthcare sector. Rather than solely being the end-users of AI systems, healthcare professionals should take a more active role in the development of frameworks for specific aspects of orthopaedic research and clinical care. Finally, expert consensus is required to integrated labelled and unlabelled orthopaedic datasets to train LLMs and GAI models to perform domain-specific tasks, such as clinical concept extraction, medical relation extraction, and medical question answering, with high efficiency, accuracy and reliability.

## Data Availability

Not applicable.

## References

[1] Eckhardt CM, Madjarova SJ, Williams RJ, Ollivier M, Karlsson J, Pareek A (2023). Unsupervised machine learning methods and emerging applications in healthcare. Knee Surg Sports Traumatol Arthrosc.

[2] Jin D, Szolovits P (2020). Advancing PICO element detection in biomedical text via deep neural networks. Bioinformatics.

[3] Karhade AV, Bongers MER, Groot OQ, Kazarian ER, Cha TD, Fogel HA (2020). Natural language processing for automated detection of incidental durotomy. Spine J.

[4] Langerhuizen DWG, Brown LE, Doornberg JN, Ring D, Kerkhoffs G, Janssen SJ (2021). Analysis of online reviews of orthopaedic surgeons and orthopaedic practices using natural language processing. J Am Acad Orthop Surg.

[5] Mesko B, Topol EJ (2023). The imperative for regulatory oversight of large language models (or generative AI) in healthcare. NPJ Digit Med.

[6] Moor M, Banerjee O, Abad ZSH, Krumholz HM, Leskovec J, Topol EJ (2023). Foundation models for generalist medical artificial intelligence. Nature.

[7] Northcutt CG, Athalye A, Mueller J (2021) Pervasive label errors in test sets destabilize machine learning benchmarks. arXiv preprint arXiv:2103.14749

[8] OpenAI (2023) GPT-4 Technical Report. https://arxiv.org/abs/2303.08774

[9] Pruneski JA, Pareek A, Kunze KN, Martin RK, Karlsson J, Oeding JF (2023). Supervised machine learning and associated algorithms: applications in orthopedic surgery. Knee Surg Sports Traumatol Arthrosc.

[10] Pruneski JA, Pareek A, Nwachukwu BU, Martin RK, Kelly BT, Karlsson J (2023). Natural language processing: using artificial intelligence to understand human language in orthopedics. Knee Surg Sports Traumatol Arthrosc.

[11] Rädsch T, Reinke A, Weru V, Tizabi MD, Schreck N, Kavur AE (2023). Labelling instructions matter in biomedical image analysis. Nat Mach Intell.

[12] Rubinger L, Gazendam A, Ekhtiari S, Bhandari M (2023). Machine learning and artificial intelligence in research and healthcare. Injury.

[13] Shiner B, Levis M, Dufort VM, Patterson OV, Watts BV, DuVall SL (2022). Improvements to PTSD quality metrics with natural language processing. J Eval Clin Pract.

[14] Singhal K, Tu T, Gottweis J, Sayres R, Wulczyn E, Hou L, et al. (2023) Towards expert-level medical question answering with large language models. arXiv preprint arXiv:2305.09617 10.1038/s41591-024-03423-7 PMC11922739 39779926

[15] Wyles CC, Tibbo ME, Fu S, Wang Y, Sohn S, Kremers WK (2019). Use of natural language processing algorithms to identify common data elements in operative notes for total hip arthroplasty. J Bone Joint Surg Am.

[16] Yang X, Chen A, PourNejatian N, Shin HC, Smith KE, Parisien C (2022). A large language model for electronic health records. NPJ Digit Med.

[17] Zhu E, Sheng Q, Yang H, Li J (2022) A Unified Framework of Medical Information Annotation and Extraction for Chinese Clinical Text. arXiv preprint arXiv:2203.03823 10.1016/j.artmed.2023.102573 37316096

[18] Zsidai B, Kaarre J, Hamrin Senorski E, Feldt R, Grassi A, Ayeni OR, et al. (2022) Living evidence: a new approach to the appraisal of rapidly evolving musculoskeletal research. Br J Sports Med. 10.1136/bjsports-2022-105570 10.1136/bjsports-2022-105570 35777954

